# Analysis of Multi-Path Fading and the Doppler Effect for Reconfigurable-Intelligent-Surface-Assisted Wireless Networks

**DOI:** 10.3390/e24020281

**Published:** 2022-02-16

**Authors:** Guilu Wu

**Affiliations:** 1 State Key Laboratory of Millimeter Waves, Southeast University, Nanjing 211189, China; guilu.wu@hainanu.edu.cn; 2 Key Laboratory of Industrial Internet of Things & Networked Control, Ministry of Education, Chongqing University of Posts and Telecommunications, Chongqing 400065, China; 3 School of Information and Communication Engineering, Hainan University, Haikou 570228, China; 4 School of Internet of Things Engineering, Jiangnan University, Wuxi 214122, China

**Keywords:** reconfigurable intelligent surface (RIS), wireless channel, multi-path fading, Doppler effect, wireless networks

## Abstract

The randomness property of wireless channels restricts the improvement of their performance in wireless networks. As a novel solution for overcoming this, a reconfigurable intelligent surface (RIS) was introduced to reshape wireless physical environments. Initially, the multi-path and Doppler effects are discussed in a case in which a reflector was considered to reflect the incident signal for wireless communication. Subsequently, the results for the transmission signal were analyzed when a reflector was coated with an RIS. Specifically, the multi-path fading stemming from the movement of the mobile transmitter was eliminated or mitigated by utilizing an RIS. Meanwhile, the Doppler effect was also reduced to restrain the rapid fluctuations in the transmission signal by using a tunable RIS in real time. The simulation results demonstrate that the magnitude and spectrum of the received signal can be regulated by an RIS. The multi-path fading and Doppler effect can be effectively mitigated when the reflector is coated with an RIS in wireless networks.

## 1. Introduction

Fifth-generation (5G) wireless networks have greater capabilities for being standardized and will be deployed worldwide in the next ten years [1]. To achieve higher goals, many unresolved problems in 5G mobile communications are expected to be solved in future wireless networks. Basically, advanced physical-layer technologies are necessary for future wireless networks [2,3]. It is noted that multi-path fading and the Doppler effect cause degradation in depictions of physical-layer technology [4,5,6,7,8]. The fundamental reason is that the wireless channel environment is random, dynamic, and uncontrollable.

As alluded to earlier, many works have studied wireless channels to improve their performance, including through advanced coding and modulation [9], multiple-input/multiple-output (MIMO) [10], cooperation communication [11], non-orthogonal multiple access (NOMA) [12], beamforming [13], array antenna [14], and much more. However, channel transmission performance has never been able to make a fundamental breakthrough with 5G technologies. RISs have played a role in controlling wireless channel environments in recent years [15]. In 2014, the concept of the RIS was proposed and verified in [16]. Its appearance builds a bridge between the physical electromagnetic (EM) world of the metasurface and the digital world of information science [17]. As a kind of metasurface, an RIS is a two-dimensional (2D) thin-layer artificial EM surface structure with reconfigurable EM properties. An RIS can use a frequency band with a range from microwave to visible light [18]. In addition, an RIS is composed of regular arrangements of EM units, which are usually composed of metal, media, and adjustable elements. The EM parameters of the reflected electromagnetic wave, such as the phase and amplitude, can be attained by controlling the EM units to enable unique EM functionalities, including phase modification, anomalous reflection, and wave regulation.

The unique EM functionalities of an RIS mentioned above could improve the performance of wireless networks by reshaping wireless channels. The authors of [19] firstly focused on analyzing the multi-path fading and Doppler effect in RIS-assisted wireless networks. In a traffic communication scenario, we further explore the potential of an RIS in suppressing the degradation of transmission performance due to multi-path fading and the Doppler effect. The distinctive contributions of this work are summarized as follows.

1. The advantages of an RIS in suppressing multi-path fading and the Doppler effect are studied with respect to a traditional reflector when the transmitter is moving along a street.
2. Considering the direct and reflected links, multi-path fading and the Doppler effect are specifically analyzed when the reflector is coated with an RIS. Specifically, the general model for the complex envelope of the combined signal at the receiver is derived in different situations.
3. A specific solution is proposed in wireless networks by utilizing an RIS to mitigate multi-path fading. Meanwhile, a solution utilizing an RIS in the presence of the Doppler effect is also provided when the direct link between the transmitter and the receiver is blocked.
4. The simulation results reveal that the multi-path fading and Doppler effect caused by the mobile transmitter can be effectively eliminated and/or mitigated by operating an RIS in real time in actual traffic applications.

In recent years, the issue of channel propagation properties has received increased attention again due to the emergence of RISs. Recently, the growing interest was mainly involved in controlling the propagation environment or exploiting its inherently random nature in order to increase the quality of service (QoS). We briefly review an overview of the state-of-the art works with RIS-assisted wireless networks that are directly relevant to our study.

In a smart radio environment, one signal is transmitted from the transmitter to the receiver [20]. The index modulation technology, which is adopted to exploit reconfigurable antenna or reflector applications in a scattering environment, is introduced to support a low complexity and a high spectrum and energy efficiency in wireless networks [21]. Against this background, many works have shown how to control wireless environments through spatial modulation [22], reflectors [23], and intelligent walls [24]. In addition, the emerging large intelligent surfaces (LISs) [25], smart reflect-arrays [26], software-defined hyperSurfaces [27], software-defined metasurfaces (SDMs) [28], passive intelligent surfaces (PISs) [29], and RISs [30] can also control impinging waves in a desired way in specific applications.

To the best of our knowledge, smart surfaces have aroused great interest in wireless networks. The related works can be classified as follows:

1. Performance optimization of the channel capacity, signal-to-noise ratio (SNR), signal-to-interference-plus-noise ratio (SINR), symbol error bits (SEBs), and energy and spectrum efficiency (EE and SE) by utilizing an RIS and transmission beamformer regulation [19,30,31].
2. Channel estimation between a BS and an RIS controller with an according phase shift for passive RIS-assisted communication to reduce training overhead [29,32,33].
3. An RIS for secure communication in a wiretap channel and by joining optimization with the transmission beamforming and RIS phase [34,35],
4. Reliability analysis of RIS-assisted communication with data-rate distribution and outage probability [36].
5. Terminal positioning and other novel applications for RIS-assisted wireless networks [29,37,38,39].

These works explored the potential of RISs from many perspectives. However, an analysis of the impact of an RIS on a wireless channel is insufficient. It is well known that multi-path fading and the Doppler effect directly affect the formation of the transmission signal. The transmission signal’s quality degrades more quickly. Fortunately, RIS technology could mitigate multi-path fading and the Doppler effect in wireless communication [40,41,42,43]. In contrast to previous traditional methods that assume that the channel has randomness and uncontrollability, the emergent concept of the RIS provides a method of reshaping the channel environment [40]. Fundamentally, multi-path fading and the Doppler effect cause a phase change in an EM wave. However, the characteristics of RISs indicate that they can improve the phase problem in EM wave transmission [41,42,43]. Hence, we further explored multi-path fading and the Doppler effect in mobile wireless environments. More importantly, the potential of RISs in terms of the improvement of multi-path fading and the Doppler effect has not yet been fully reported. In light of this, the potential of RISs in terms of multi-path fading and the Doppler effect is revisited by considering a traffic scenario. Specifically, the received signal strength is analyzed when a reflector or RIS is deployed in a wireless network. The received combined signals in two cases are analyzed to dig out their relationship with and without RIS-assisted communication. An RIS’s mitigation of multi-path fading when it is coated onto a reflector is discussed. Meanwhile, the Doppler effect can also be effectively restrained by controlling an RIS in real time. All results reveal that RISs have great potential to improve the performance of wireless channel environments.

The structure of the rest of the article is organized as follows. In Section 2, we describe vehicle traffic at a crossroads and build mathematical models of multi-path fading and the Doppler effect stemming from the movement of a mobile vehicle while considering a reflector and an RIS, respectively. In addition, a solution for the suppression of the Doppler effect with an RIS is explained in Section 3. The simulation results in Section 4 display the multi-path fading and Doppler effect caused by the movement of a mobile transmitter under a traffic propagation scenario (with and without an RIS). Section 5 contains discussions of practical issues and future work on applications with RISs. Finally, the conclusions are summarized in Section 6.

## 2. Analysis of Multi-Path Fading and the Doppler Effect with a Traffic Scenario

In this section, the multi-path fading and Doppler effect are analyzed in mobile communication systems with and without an RIS. The signal transmission waveform is focused on the case of a low-pass equivalent and noise-free communication.

### 2.1. Description of the System Model

**Figure 1:**
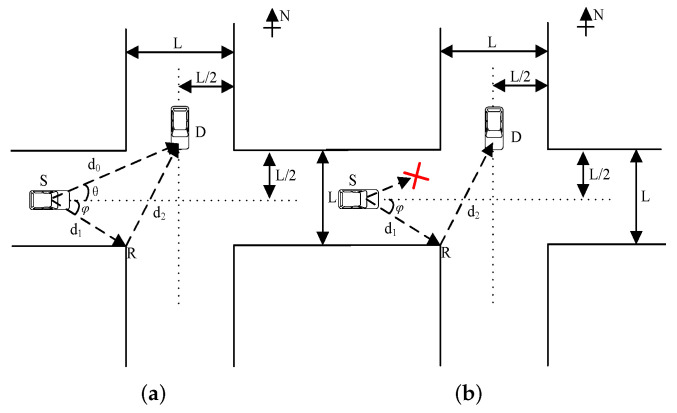
An example of a geometric diagram with vehicles’ communications for S→R→D when the link S→D is normal or blocked (S↛D). (**a**) Geometric diagram. (**b**) Geometric diagram without LOS.

In a traffic scenario, a vehicle is driving through the intersection at speed *v* from west to east along the road, as shown in Figure 1a. To improve the safety of driving the vehicle at traffic intersections, this vehicle transmits a signal to a waiting vehicle located in the vertical direction. Reflection on the wall interferes with the transmission signal. To ensure the quality of the communication link, one reflection point (a reflector or an RIS) *R*, which provides an additional communication link, is deployed on the side of the corner near the intersection. To make this article easy to read, some definitions of symbols are summarized in Table 1.

In Figure 1a, a cartesian coordinate system is established with the center of the intersection being denoted as (0,0). The two streets have the same width *L*. The source vehicle *S* at location (x,0), $x\leq -\frac{L}{2}$, transmits signals to the destination vehicle *D*, which is located at (0,L/2). A reflector or an RIS is deployed on the reflection point, *R*, which is at $\left(-\frac{L}{2},-\frac{L}{2}\right)$. Hence, vehicle *D* can receive signals from the links S→D and S→R→D. Meanwhile, the angles between S→D and S→R and the horizontal line are θ and φ, respectively. The distances between *S*, *D*, and *R*, $d_{0}$, $d_{1}$, and $d_{2}$, and they vary with the time *t* due to the movement of vehicle *S*, which is given by

(1) $$\left\{\begin{array}{cc} d_{0} & =\sqrt{x^{2}+{\left(\frac{L}{2}\right)}^{2}},\\ d_{1} & =\sqrt{{\left(x+\frac{L}{2}\right)}^{2}+{\left(\frac{L}{2}\right)}^{2}},\\ d_{2} & =\frac{\sqrt{5}}{2}L, \end{array}\right.$$

and

(2) $$\left\{\begin{array}{cc} \theta & =\arcsin\left(\frac{L}{2x}\right),\\ \varphi & =\arcsin\left(\frac{L}{2x-L}\right), \end{array}\right.$$

We assume that vehicle *S* transmits a radio-frequency (RF) carrier signal u(t), $u\left(t\right)=a\left(t\right)\cos\left(2\pi f_{c}t-k_{c}z+\phi_{0}\right)$, where a(t) is the envelope of u(t), $f_{c}$ is the carrier frequency, *z* is the distance along the propagation direction of the EM wave, and $\phi_{0}$ is the initial phase. The propagation coefficient $k_{c}$ is defined as

(3) $$k_{c}=2\pi /\lambda_{c},$$

where $\lambda_{c}$ is the EM wavelength. Because a signal can be represented by the envelope and phase, which is also called a complex-envelope signal, the low-pass equivalent of signal u(t) is expressed as [7,44]

(4) $$r\left(t\right)=a\left(t\right)\exp\left(-jk_{c}z\right),$$

where the initial phase has been dropped for convenience, i.e., $\phi_{0}=0$. We denote with d(t) the distance between the transmitter and the receiver at time *t*. Thus, (4) can be rewritten as

(5) $$r\left(t\right)=a\left(t\right)\exp(-jk_{c}d\left(t\right)),$$

where $a\left(t\right)=\lambda_{c}/4\pi d(t)$. It is noted that the antenna has omnidirectional and unit gain in linear units for free-space propagation.

The propagation distance of a signal constantly varies with the movement of vehicle *S*. Given the initial distance *d*, we can derive d(t) from

(6) d(t)=d−Vt,

where $V_{S\to D}=v\cos\theta$ and $V_{S\to R}=v\cos\varphi$ indicate the speed in the links S→D and S→R, respectively.

We have

(7) $$\left\{\begin{array}{cc} f_{d} & =\frac{v}{\lambda_{c}}\cos\theta =f_{c}\frac{v}{c}\cos\theta =f_{m_{d}}\cos\theta ,\\ f_{r} & =\frac{v}{\lambda_{c}}\cos\varphi =f_{c}\frac{v}{c}\cos\varphi =f_{m_{r}}\cos\varphi , \end{array}\right.$$

where *c* is the speed of light. When θ=0 and φ=0, $f_{m_{d}}$ and $f_{m_{r}}$ are the maximum values with respect to the carrier frequency $f_{c}$, respectively. Hence,

(8) $$f_{m_{d}}=f_{m_{r}}=f_{c}\frac{v}{c}.$$

### 2.2. Multi-Path Fading and the Doppler Effect with a Reflector

We consider a short stretch of travel distance before vehicle *S* crosses the intersection. Vehicle *D* receives two signals, one from the S→D link and the other from the S→R→D link (*R* represents the reflection point at which a reflector or an RIS is deployed; their difference is not the focus of this paper). The reflection coefficient on *R* is σ, which depends on the reflection materials. The signal received on vehicle *D* has a constant amplitude, but displays a rapidly changing phase. The signals received from S→D and S→R→D can be expressed as

(9) $$r_{c}\left(t\right)=r_{\left\{S\to D\right\}}\left(t\right)+\sigma \cdot r_{\left\{S\to R\to D\right\}}\left(t\right),$$

Specifically, (9) can be rewritten as

(10) $$r_{c}\left(t\right)=\frac{\lambda_{c}}{4\pi }\left(\frac{e^{-j\frac{2\pi }{\lambda_{c}}d_{0}\left(t\right)}}{d_{0}\left(t\right)}+\sigma \frac{e^{-j\frac{2\pi }{\lambda_{c}}\left(d_{1}\left(t\right)+d_{2}\left(t\right)\right)}}{d_{1}\left(t\right)+d_{2}\left(t\right)}\right),$$

where

(11) $$\left\{\begin{array}{cc} d_{0}\left(t\right) & =d_{0}-vt\cos\theta ,\\ d_{1}\left(t\right) & =d_{1}-vt\cos\varphi ,\\ d_{2}\left(t\right) & =d_{2}, \end{array}\right.$$

We assume that the signal from vehicle *S* has a constant amplitude in the communication process. In this case, (10) can be simplified to

(12) $$r_{c}\left(t\right)=\frac{\lambda_{c}}{4\pi }\left(\frac{e^{-j2\pi f_{d}t\cos\theta -j\phi_{d}}}{d_{0}}+\sigma \frac{e^{-j2\pi f_{r}t\cos\varphi -j\phi_{r}}}{d_{1}+d_{2}}\right),$$

where $\phi_{d}=2\pi d_{0}/\lambda_{c}$ and $\phi_{r}=2\pi \left(d_{1}+d_{2}\right)/\lambda_{c}$. $f_{d}\cos\theta$ and $f_{r}\cos\varphi$ are Doppler shifts with regard to $f_{c}$ Hz or 0 Hz in the low-pass equivalent or pass-band signal, respectively. Without loss of generality, the initial phase ϕ can be dropped when it follows that ϕ=2kπ, k∈Z. The specific analyses of the amplitude value, $|r_{c}\left(t\right)|$, and the phase value, $∠r_{c}\left(t\right)$, are outlined in Appendix A. From complex exponential theory, the magnitude of (12) is derived as

(13) $$\begin{array}{cc} |r_{c}\left(t\right)|= & \frac{\lambda_{c}}{4\pi }(\frac{1}{d_{0}^{2}}+{\left(\frac{\sigma }{d_{1}+d_{2}}\right)}^{2}+\frac{2\sigma }{d_{0}\left(d_{1}+d_{2}\right)}\\ \; & \cdot \cos\left(2\pi \left(f_{r}\cos\varphi -f_{d}\cos\theta \right)t\right){)}^{\frac{1}{2}}. \end{array}$$

and the phase value is

(14) $$\begin{array}{c} ∠r_{c}\left(t\right)=-\arctan^{-1}\tan\left(\frac{\left(d_{1}+d_{2}\right)\sin\Lambda +\sigma d_{0}\sin\Gamma }{\left(d_{1}+d_{2}\right)\cos\Lambda +\sigma d_{0}\cos\Gamma }\right). \end{array}$$

where $\Lambda =2\pi f_{d}t\cos\theta$, $\Gamma =2\pi f_{r}t\cos\varphi$.

It is noted that the value of the reflection coefficient, σ, is related to the transmission direction of the reflected wave. Ideally, the reflection coefficient σ is one, and the phase is zero. Otherwise, the phase of the reflection coefficient σ is π.

### 2.3. Multi-Path Fading and the Doppler Effect with an RIS

When the reflection point *R* is coated on an RIS, it can be represented by a time-varying gain-reflection coefficient, that is, $\sigma \left(t\right)=\beta e^{j\Theta (t)}$, β∈[0,1], Θ(t)∈[0,2π). Then, the complex envelope of the signal received at vehicle *D* can be expressed as

(15) $$r_{c}\left(t\right)=\frac{\lambda_{c}}{4\pi }\left(\frac{e^{-j2\pi f_{d}t\cos\theta }}{d_{0}}+\beta \frac{e^{-j(2\pi f_{r}t\cos\varphi -\Theta \left(t\right))}}{d_{1}+d_{2}}\right),$$

If the magnitude is at its maximum or minimum, the phase in the reflection coefficient, σ(t), is described as

(16) $$\Theta \left(t\right)=2\pi t\left(f_{r}\cos\varphi -f_{d}\cos\theta \right)+2k\pi ,k\in Z,$$

or

(17) $$\Theta \left(t\right)=2\pi t\left(f_{r}\cos\varphi -f_{d}\cos\theta \right)+\left(2k+1\right)\pi ,k\in Z.$$

In other words, the phases of the EM wave signals of S→D and S→R→D are fully aligned according to (16), while (17) indicates that their phases have opposite directions. A specific analysis will be discussed in the following.

#### 2.3.1. Eliminating Multi-Path Fading and the Doppler Effect with an RIS

Due to technical limitations, the phase alignment of a two-ray EM wave signal is challenging in practice. Generally, Θ(t) is constant at discrete-time instants. From the discussion of Θ(t), the complex envelope of $r_{c}\left(t\right)$ can, therefore, be expressed as

(18) $$r_{c}\left(t\right)=\frac{\lambda_{c}}{4\pi }e^{-j2\pi f_{d}t\cos\theta }\left(\frac{1}{d_{0}}+\frac{\beta }{d_{1}+d_{2}}\right).$$

The maximum amplitude is given by

(19) $$|r_{c}\left(t\right){|}_{max}=\frac{\lambda_{c}}{4\pi }\left(\frac{1}{d_{0}}+\frac{\beta }{d_{1}+d_{2}}\right).$$

#### 2.3.2. Utilizing Multi-Path Transmission with an RIS

Considering a case in which the signal may be received by illegal users, such as eavesdroppers, to solve this problem, we reduce the strength of the received signal for the illegal user by utilizing multi-path fading and the Doppler effect in an RIS-assisted wireless network. As a result, the complex envelope of $r_{c}\left(t\right)$ on vehicle *D* is given by

(20) $$r_{c}\left(t\right)=\frac{\lambda_{c}}{4\pi }e^{-j2\pi f_{d}t\cos\theta }\left(\frac{1}{d_{0}}-\frac{\beta }{d_{1}+d_{2}}\right).$$

In addition, the minimum magnitude of $r_{c}\left(t\right)$ is given by

(21) $$|r_{c}\left(t\right){|}_{min}=\frac{\lambda_{c}}{4\pi }\left(\frac{1}{d_{0}}-\frac{\beta }{d_{1}+d_{2}}\right).$$

## 3. Suppression of the Doppler Effect with an RIS

The Doppler effect will be analyzed in the following for when the communication link S↛D is blocked, as shown in Figure 1b. The communication link between vehicles *S* and *D* is established through an RIS. The same assumptions as in Section 2 are defined to investigate and analyze the Doppler effect in different cases.

### 3.1. Communication Link S→R→D with a Reflector

Similarly, the signal received on vehicle *D* with a reflection coefficient σ for reflector *R* can be expressed as

(22) $$r_{c}\left(t\right)=\sigma \frac{\lambda_{c}}{4\pi }\cdot \frac{e^{-j\frac{2\pi }{\lambda_{c}}\left(d_{1}\left(t\right)+d_{2}\left(t\right)\right)}}{d_{1}\left(t\right)+d_{2}\left(t\right)},$$

where $d_{1}\left(t\right)$ varies with the movement of vehicle *S* at a speed *v*. Considering a short distance, the constant term on the phase is ignored in the received signal for vehicle *D*. Then, the result is

(23) $$r_{c}\left(t\right)=\sigma \frac{\lambda_{c}}{4\pi }\cdot \frac{e^{-j2\pi f_{r}t\cos\varphi }}{d_{1}\left(t\right)+d_{2}\left(t\right)}.$$

In terms of frequency shift, the signal received on vehicle *D* still has a Doppler shift, $f_{r}\cos\varphi \;Hz$. Furthermore, the signal received on vehicle *D* via *R* cannot display a fading pattern due to dropping a multi-path component. Hence, the received signal has a fixed amplitude value with respect to time, and it is given by

(24) $$|r_{c}\left(t\right)|=\frac{\sigma \lambda_{c}}{4\pi (d_{1}\left(t\right)+d_{2}\left(t\right))}.$$

### 3.2. Communication Link S→R→D with an RIS

An RIS is mounted on the reflection point, *R*, and its reflection coefficient is defined as $\sigma \left(t\right)=\beta e^{j\Theta (t)}$. Then, we have

(25) $$r_{c}\left(t\right)=\beta \frac{\lambda_{c}}{4\pi }\cdot \frac{e^{-j(2\pi f_{r}t\cos\varphi -\Theta \left(t\right))}}{d_{1}\left(t\right)+d_{2}\left(t\right)}.$$

With respect to (23) and (25), they have the same amplitude value. This indicates that an RIS cannot affect the magnitude of the received signal for vehicle *D*. However, it could eliminate the Doppler effect completely if the Θ(t) satisfies

(26) $$\Theta \left(t\right)=-2\pi f_{r}t\cos\varphi +2k\pi ,k\in Z.$$

## 4. Simulation Results

According to the system model in Section 2, the performance of the received signal with respect to multi-path fading and the Doppler effect in a mobile wireless environment is analyzed. In this case, the received signal captured by reflectors at the receiving terminal is investigated to analyze the network deployment for multiple network parameters, such as network geometry, transmission location, the number of reflectors or RISs, etc. An experimental scenario was developed in Matlab to simulate the *R*-assisted communication. Vehicle *S* is driving through an intersection on a street with width L=40 m at speed v=10 m/s. The carrier frequency of the transmission signal is $3\times 10^{9}$ (Hz). For the convenience of the experiment, different starting positions of vehicle *S* before passing the intersection were used as experimental subjects, taking, for example, |x|=1000,800,600,400 m.

The two cases of *R* representing a reflector or an RIS were individually considered when vehicle *S* transmits a signal to vehicle *D* in a wireless network. Their corresponding experimental results will be given and discussed in the following.

### 4.1. Case of *R* as a Reflector

Initially, vehicle *D* receives two signals, which are from links S→D and S→R→D, depending on the geometry, as shown in Figure 1a. Here, for convenience, we assume that the reflector *R* has ideal material conditions, and the reflection coefficient is σ=1.

**Figure 2:**
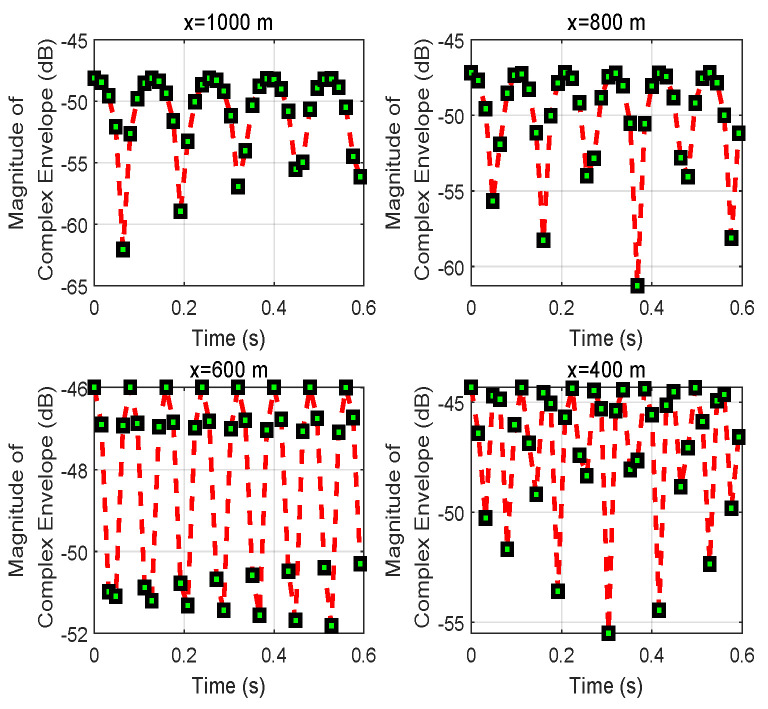
Variation of the magnitude of the complex envelope received at vehicle *D* for different initial positions *x* when the speed of vehicle *S* is *v*.

In Figure 2, the magnitude of the complex envelope of the combined signal at vehicle *D* when vehicle *S* travels a short distance is plotted. The results are for different initial positions |x| of vehicle *S*: x=1000,800,600,400 m. Clearly, the magnitude of the complex envelope shows rapid fluctuations around a mean value at different times. This is because the two arriving signals easily produce detrimental and beneficial interactions. In addition, this process is also affected by path loss. Furthermore, the oscillation of the received signal can be seen as the fading pattern of the multi-path transmission. It is worth noting that the magnitude of the received signal is more significant for larger values of *x* that satisfy $d_{0}\approx d_{1}+d_{2}$.

**Figure 3:**
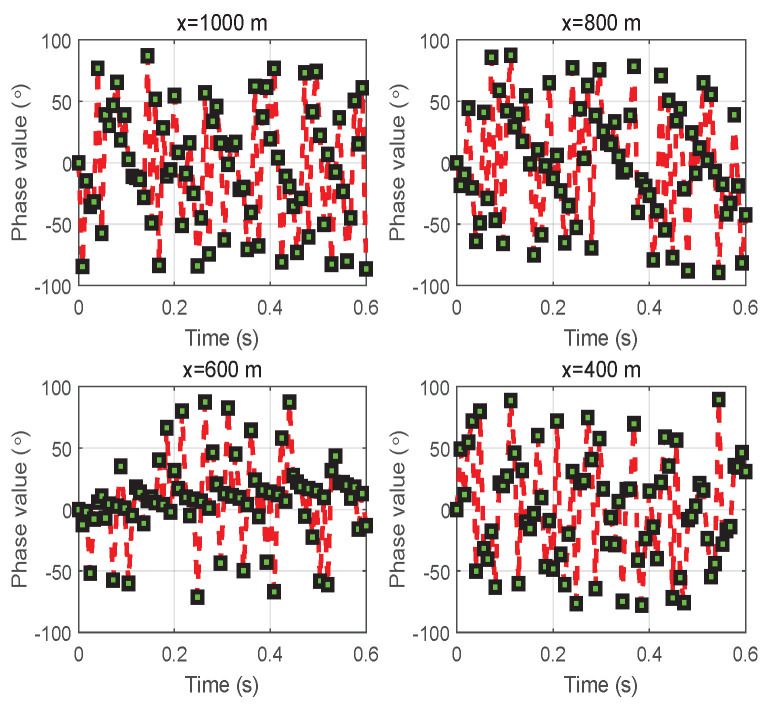
Variation of the phase value of the combined signal received at vehicle *D* for different initial positions *x* when the speed of vehicle *S* is *v*.

Correspondingly, the phases of the combined signal at vehicle *D* for different vehicle positions are displayed in Figure 3. The phase when spreading in degrees in the interval [−90,90] varies with the movement of vehicle *S* in real time. This can be verified with Figure 1a in a real scenario. During the movement of vehicle *S*, the phase changes with the change in distance from $d_{0}$ to $d_{1}$.

**Figure 4:**
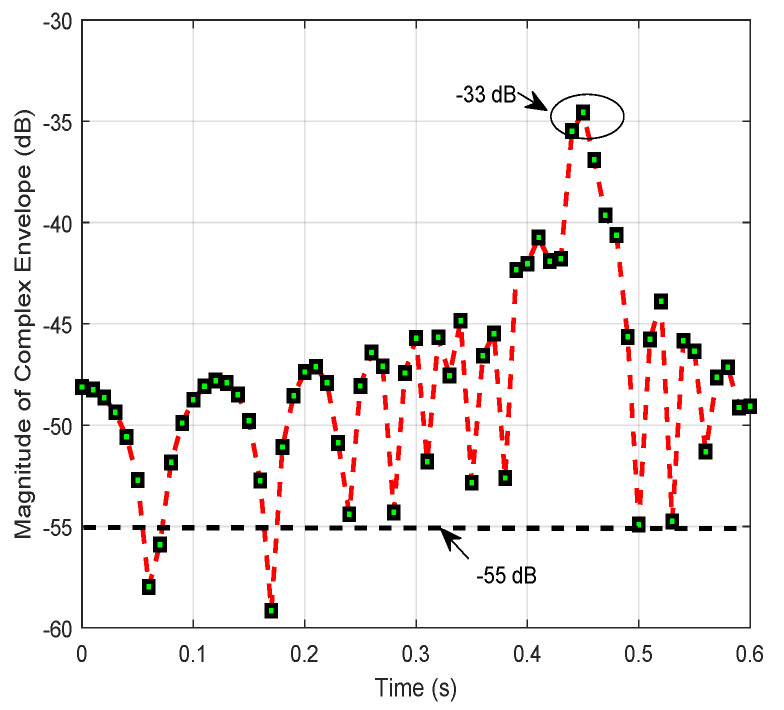
The variation process of the magnitude of the complex envelope received at vehicle *D* from vehicle *S* with v=10 m/s for the initial position x(−1000,0) m.

Specifically, taking |x|=1000 as an example, vehicle *S* drives from the initial position (−1000,0) m. The magnitude of the complex envelope of the combined signal at vehicle *D* is plotted in Figure 4. The result undergoes significant changes with ups and downs at time 0.44 s. Otherwise, the result reaches the peak at about −33 dB before dropping considerably to an average volatile value of −55 dB. In addition, the fluctuation of the signal at the initial and final stages appears significantly different due to the different propagation distances of $d_{0}$ and $d_{1}+d_{2}$.

**Figure 5:**
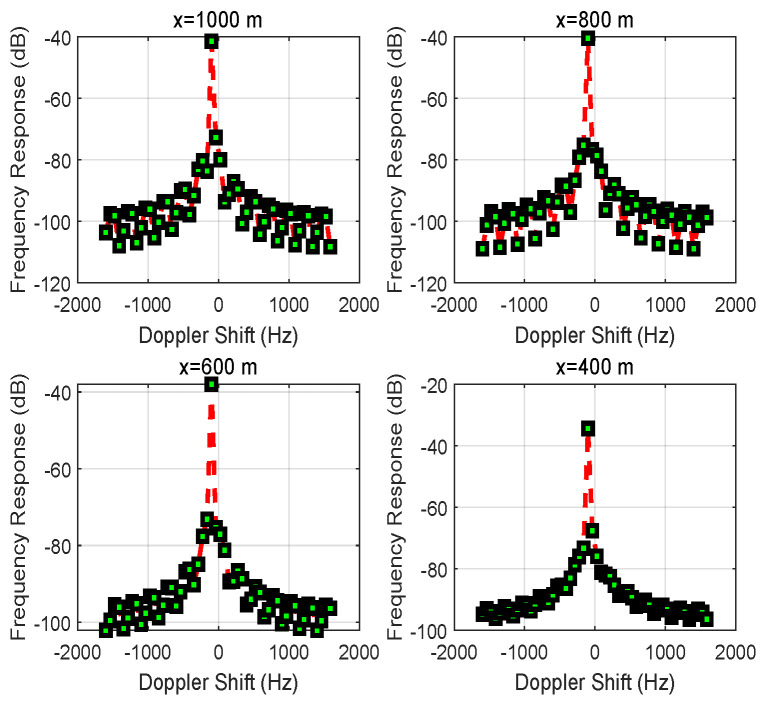
The frequency response of the signal received at vehicle *D* when changing the position *x* of vehicle *S*.

Figure 5 shows how the frequency response varies with the Doppler shifts based on the different initial distances |x| for vehicle *S*. With different amplitudes and different transmission distances in two directions (i.e., S→D and S→R→D), the sharp component of the Doppler shift is closer to 0 Hz than to the negative side. Overall, the oscillation slows down from x=1000 to 400 m due to the path loss caused by the multi-path transmission.

**Figure 6:**
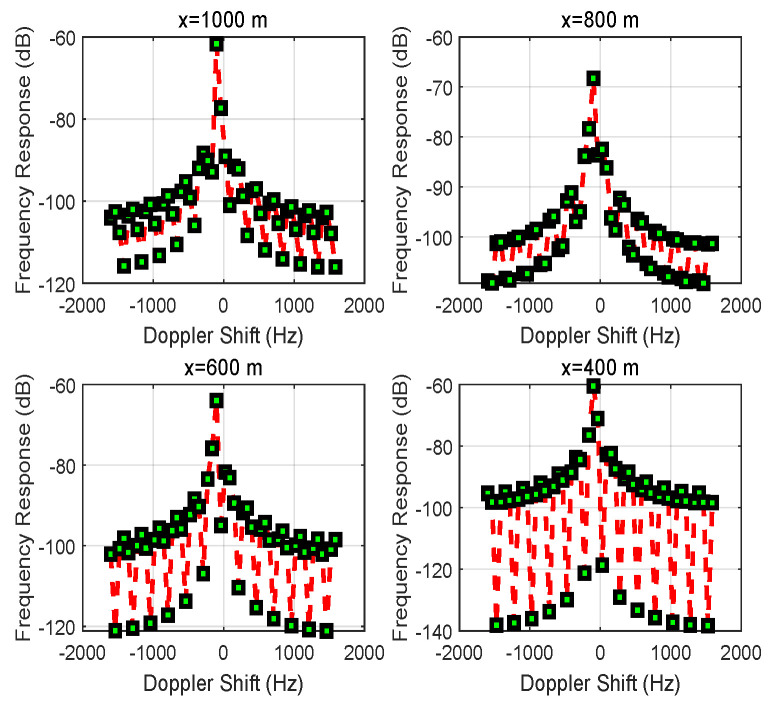
The frequency response of the received signal for different positions *x* of vehicle *S* for the case of S↛D.

In Figure 1b, the Doppler effect from Figure 6 can be observed, which shows the frequency response of the received signal for different initial distances |x| of vehicle *S*. The result indicates that the received signal’s strength is enhanced in terms of frequency response as the multi-path fading drops. However, the movement of vehicle *S* causes the carrier frequency to deviate from 0 Hz (Doppler shift).

### 4.2. *R* as an RIS

**Figure 7:**
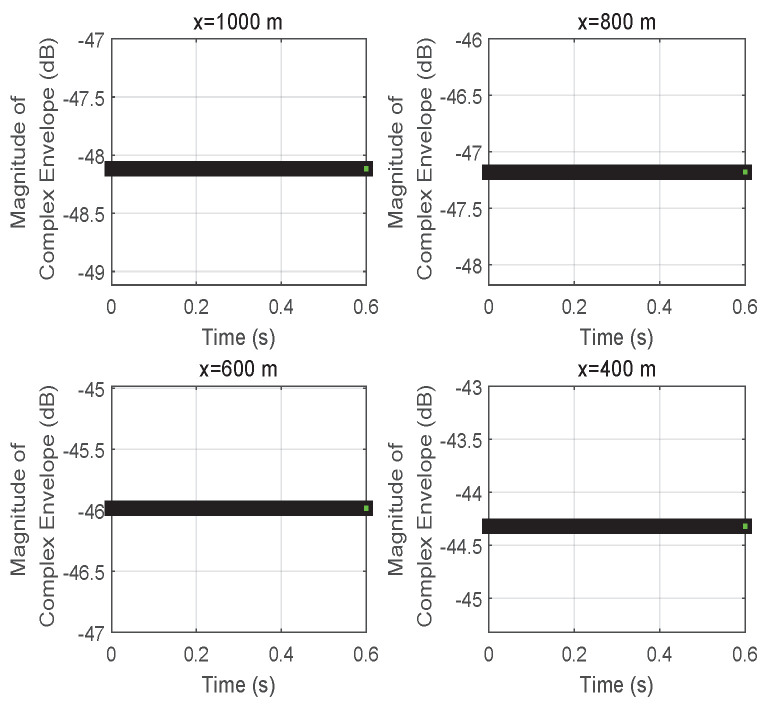
The magnitude of the complex envelope received at vehicle *D* for different initial positions *x* of vehicle *S*.

**Figure 8:**
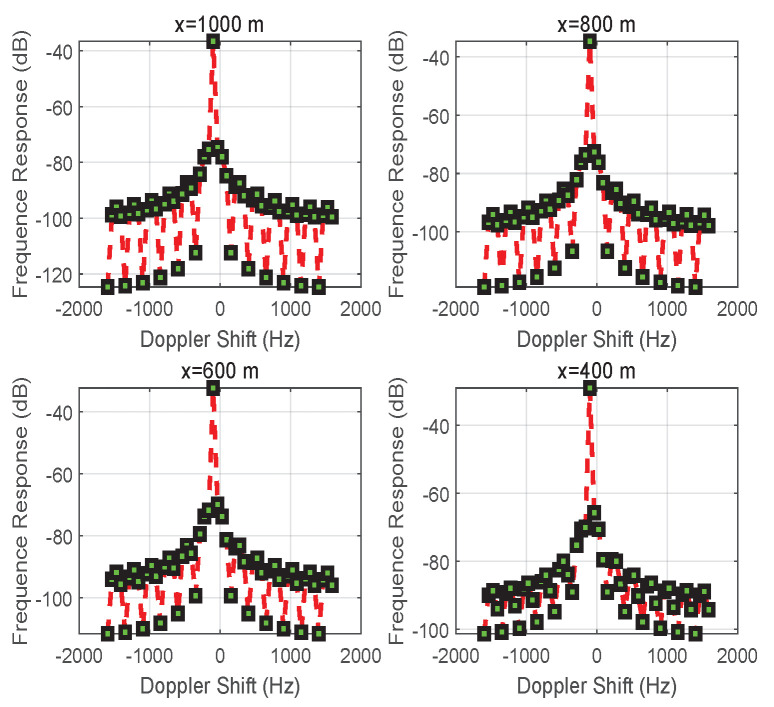
The frequency response of the received signal at different positions *x* by utilizing an RIS to eliminate multi-path fading.

When the reflector is coated with an RIS, the propagation environment can be controlled by designing the reflection coefficient σ of an RIS to satisfy the requirements of different applications in RIS-assisted wireless networks. For this case, the magnitude of the complex envelope of the received signal at vehicle *D* is given in Figure 7. Compared with Figure 2, the rapid volatility of the combined signal due to multi-path propagation is eliminated in Figure 7. The reason is that the controllability of an RIS can regulate the EM wave from the movement of vehicle *S* in real time. However, the Doppler effect always accompanies the communication process between vehicles *S* and *D*, as shown in Figure 8. The signal received at vehicle *D* is subject to Doppler shift (it is noted that the Doppler shift is determined by both $f_{d}$ Hz and $f_{r}$ Hz from (7)). Although the RIS can effectively eliminate multi-path fading, it cannot avoid a Doppler frequency shift in this case. The additional frequency shift of which the RIS loses control is caused by the communication link between vehicles *S* and *D*.

**Figure 9:**
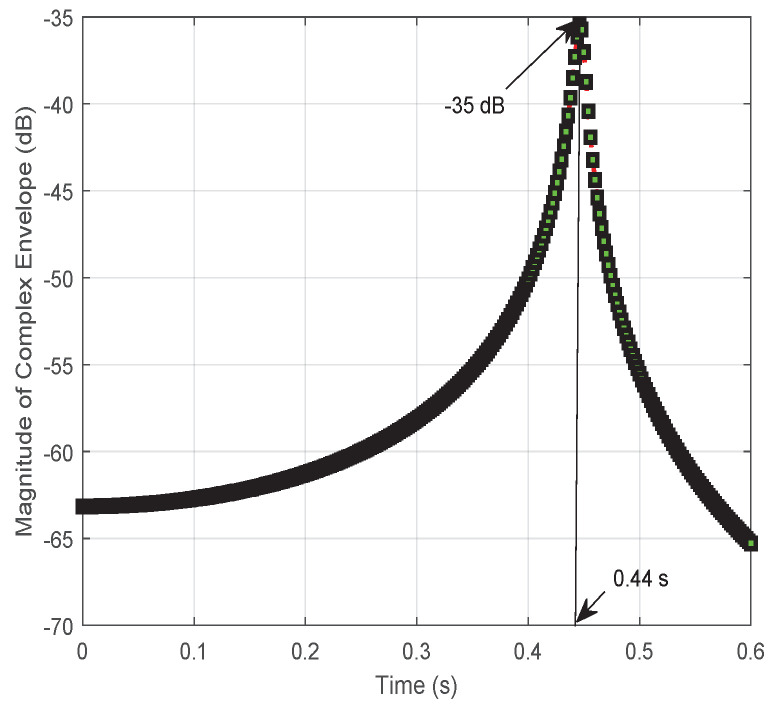
The variation process of the magnitude of the complex envelope received at vehicle *D* when vehicle *S* is moving at a speed of *v* from the initial position x=1000 m.

For the initial position |x|=1000 m, the variation process of the magnitude of the complex envelope at different times while vehicle *S* is driving is demonstrated in Figure 9. In more detail, the continuous changes in transmission distance at different times cause changes in the magnitude of the complex envelope for the received signal. Initially, the result slowly reaches a peak at −35 dB at time 0.44 s, and then encounters an abrupt decrease later in the observation range [0,0.6] s.

**Figure 10:**
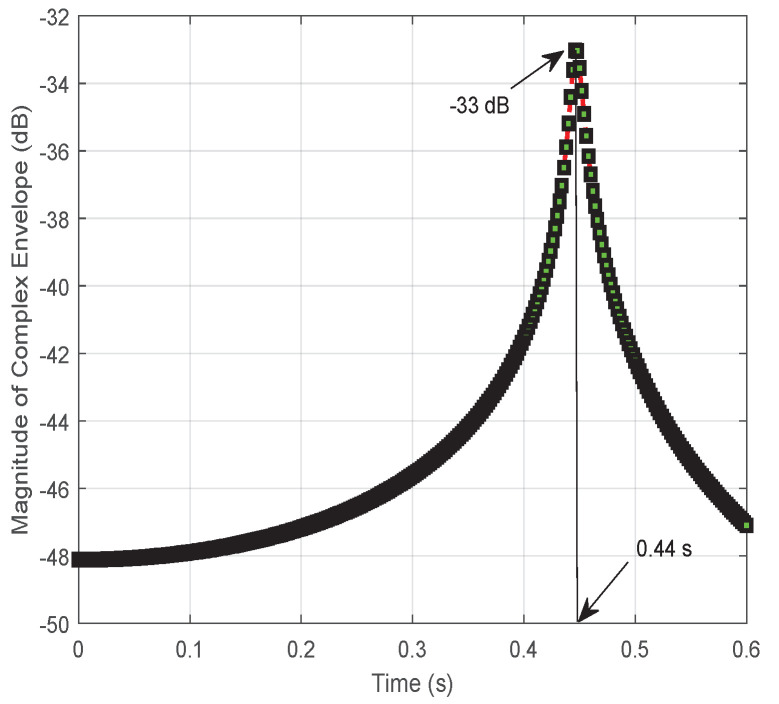
The variation process of magnitude of the received complex envelope when vehicle *D* moves from initial position x=1000 m under minimizing the received signal by utilizing an RIS.

**Figure 11:**
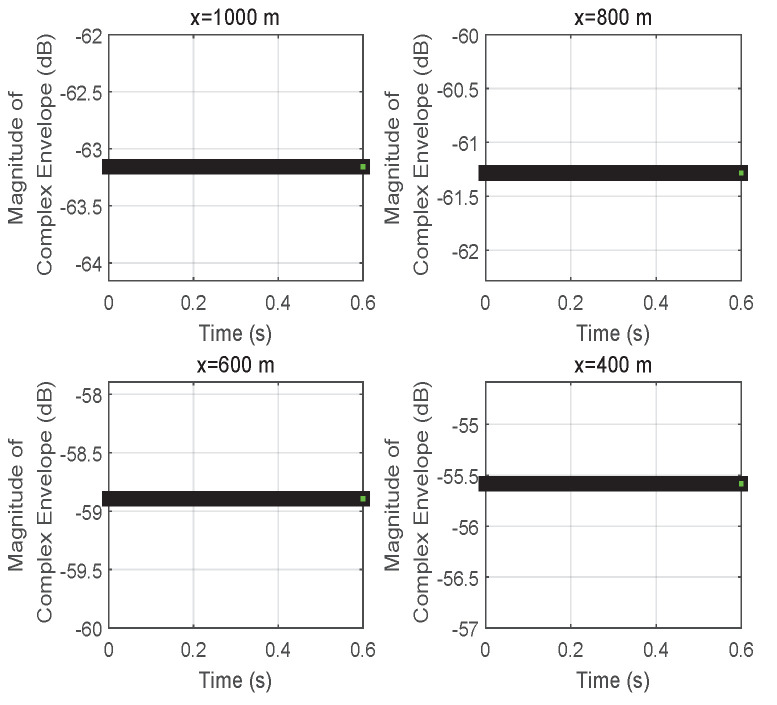
The magnitude of the complex envelope received at vehicle *D* for different initial positions *x* of vehicle *S* when minimizing the received signal by utilizing an RIS.

**Figure 12:**
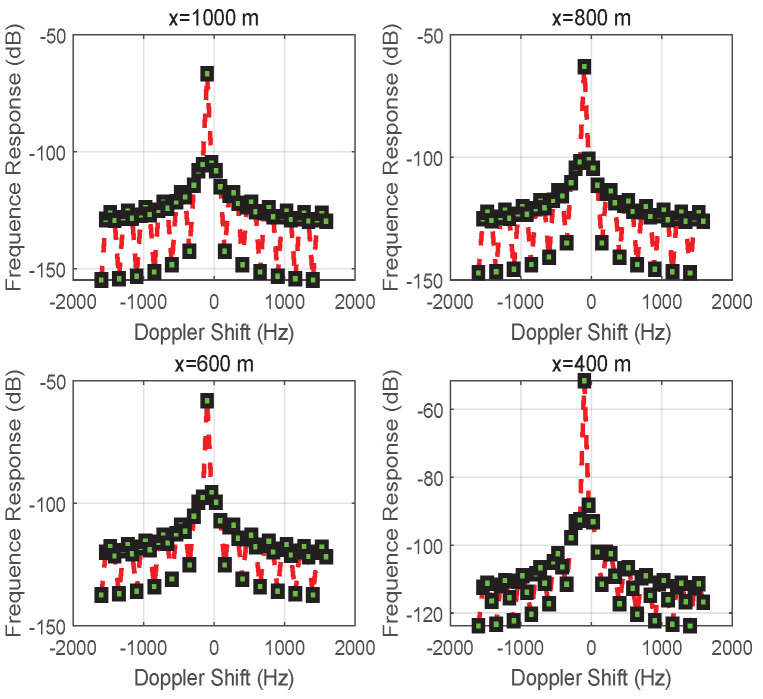
The frequency response of the received signal for different positions *x* while utilizing an RIS to increase multi-path fading and the Doppler effect.

In Figure 10, Figure 11 and Figure 12, the results focus on the maximization of the signal received on vehicle *D* by controlling the reflection phase of an RIS in real time. However, the Doppler effect is increased to degrade the received signal’s strength when the transmission signal may be intercepted by an unintended receiving terminal or an eavesdropper. As we said before, when the signals received on vehicle *D* from S→D and S→R→D are aligned according to (18), the maximum magnitude for the signal received on vehicle *D* can be obtained with (19). However, if the received signals are completely out of phase, thus satisfying (17), the signal strength on vehicle *D* is degraded to (21) based on (20), especially for $d_{0}\approx d_{1}+d_{2}$ and β=1. The vivid results on the magnitude of the complex envelope have been depicted in Figure 11 for varying distances *x*. A degradation in magnitude of 15 dB happens at x=1000 m when comparing Figure 7 with Figure 11. Meanwhile, the variation process of the magnitude of the complex envelope received at different times when vehicle *S* moves from the position x=1000 m is given in Figure 10. At this time, the received complex envelope is about −33 dB at time 0.44 s for the phase opposite to the received combined signals.

**Figure 13:**
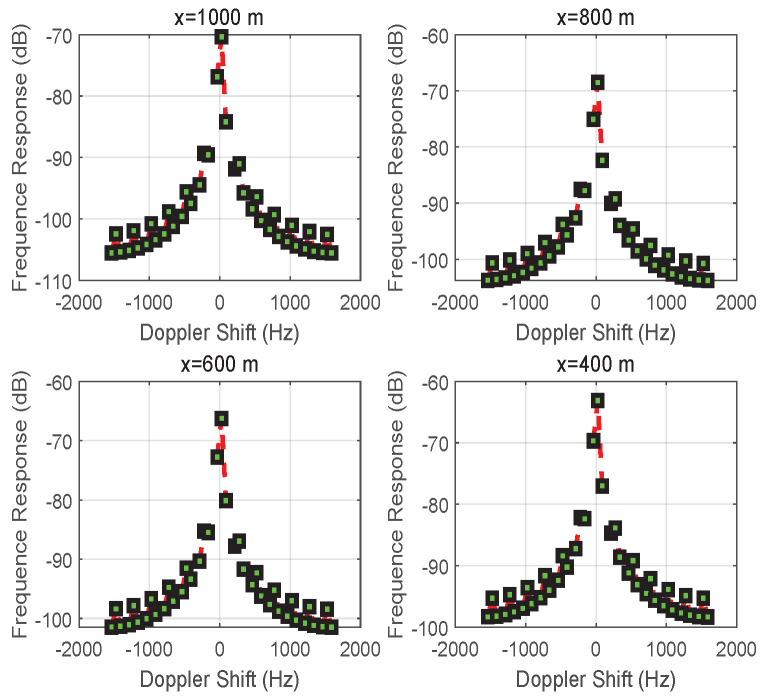
The frequency response of the received signal for different positions *x* of vehicle *S* while utilizing an RIS to eliminate Doppler shift for the case of S↛D.

When the link between vehicles *S* and *D* is blocked, S↛D, the Doppler effect is verified by controlling an RIS, as shown in Figure 1b. Figure 13 shows the frequency response of the received signal for different positions |x| of vehicle *S*, and the Doppler shift is 0 Hz.

## 5. Existing Practical Issues and Future Work

In this section, we focus on some practical problems in RIS-assisted wireless networks. A number of perfect conditions were given in our models and solutions. Future work is also discussed in this part.

### 5.1. Existing Practical Issues

Regarding the choice of an RIS, we assumed that the reflection coefficient of an RIS has a unit amplitude of β=1 and a tunable full-positive phase in real time, Θ∈[0,2π). However, the reflection coefficient of an RIS involves not only the amplitude and phase mentioned, but also the physical size from the perspective of the vehicle. Hence, we should consider a limited size of the RIS.

In addition, we considered that vehicle *S* is driving at speed v=10 m/s in a vehicular network and that the reflection phase of the RIS has discrete values. Hence, we can assume that the reflection phase of the RIS remains constant during a period. Furthermore, as discussed earlier, our models and solutions are also valid for high-mobility scenarios in which the reflection phase of the RIS can be tuned in real time at a proper rate. However, it is difficult to control the phase of an RIS in real time. This is limited by the design of the RIS and the amount of signal interaction overhead in wireless networks. In most cases, the reflection phase of an RIS can be controlled at certain discrete times. In real life, the vehicles and RISs remain at the same level when reducing transmission distance error. Furthermore, the RIS should stay in sync with the communication devices. In this case, the reflection phases of the RIS can be tuned in real time with communication devices at a sufficiently high regulation rate.

### 5.2. Future Works

In this paper, we focus on multi-path fading pattern and Doppler shift in a two-way transmission scenario. A discussion of a case with more reflective surfaces is missing. This paper only considers a channel propagation environment with multi-path fading and the Doppler effect. On this basis, the imperfect conditions of networks affect their performance, such as in practical magnitude and phase, discrete-time phase, erroneous estimation, interference, and so on. In addition, this scenario was very simple, as it only included one-way traffic and two vehicles.

Furthermore, research on RIS-assisted wireless networks is still in the early stages of development. We can explore some other interesting research issues, such as reflection coefficient modulation, a practical propagation model, a path-fading model for complex scenarios, and so on.

## 6. Conclusions

To sum up, we discussed multi-path fading and the Doppler effect for reflector-/RIS-assisted wireless networks. Specifically, we revisited multi-path fading and the Doppler effect in mobile wireless networks, and corresponding solutions for eliminating Doppler shift by utilizing an RIS were also proposed. In this regard, simple scenarios with or without an RIS were considered. Then, we deduced the models of the received signal to consider multi-path fading and the Doppler effect. In addition, we proposed solutions for obtaining the maximum and minimum magnitude conditions by tuning the reflection phase of the RIS. Furthermore, the elimination and utilization of multi-path fading and the Doppler effect with an RIS were analyzed for special applications. The suppression Doppler effect was described in a scenario in which the direct link was blocked. Finally, simulation results from our novel models and solutions in wireless networks were discussed. 

## Figures and Tables

**Table 1:** A summary of the definitions of symbols.

Symbol	Definition
*S*	Source vehicle
*D*	Destination vehicle
*R*	Reflector or RIS
*L*	Street width
|x|	Initial position of *S*
$d_{0}$	Distance between *S* and *D*
$d_{1}$	Distance between *S* and *R*
$d_{2}$	Distance between *R* and *D*
θ	Angle between S→D and a horizontal line
φ	Angle between the horizontal line and S→D
$k_{c}$	Propagation coefficient
$f_{c}$	Carrier frequency
$f_{d}$ and $f_{r}$	Doppler shift
λ	Carrier wavelength
*v*	Vehicle speed of *S*
σ and β	Reflection coefficient of *R*
$r_{c}$	Complex envelope of the signal
$|r_{c}|$	Magnitude of $r_{c}$
Θ	Phase of the RIS

## Appendix A

We define $z_{1}=r_{1}e^{j\omega_{1}}$, $z_{2}=r_{2}e^{j\omega_{2}}$, and $z_{3}=r_{3}e^{j\omega_{3}}$, and we point out the following relationship: $z_{3}=z_{1}+z_{2}$.

Therein,

(A1) $$\begin{array}{cc} z_{3} & =z_{1}+z_{2}\\ \; & =r_{1}e^{j\omega_{1}}+r_{2}e^{j\omega_{2}}\\ \; & =a+bj, \end{array}$$

where $a=r_{1}\cos\omega_{1}+r_{2}\cos\omega_{2}$ and $b=r_{1}\sin\omega_{1}+r_{2}\sin\omega_{2}$.

Then,

(A2) $$\begin{array}{cc} |r_{3}| & =\sqrt{a^{2}+b^{b}}\\ \; & =\sqrt{{\left(r_{1}\cos\omega_{1}+r_{2}\cos\omega_{2}\right)}^{2}+{\left(r_{1}\sin\omega_{1}+r_{2}\sin\omega_{2}\right)}^{2}}\\ \; & ={\left(r_{1}^{2}+r_{2}^{2}+2r_{1}r_{2}\cos\left(\omega_{1}-\omega_{2}\right)\right)}^{\frac{1}{2}}, \end{array}$$

and

(A3) $$\begin{array}{cc} \omega_{3} & =\arctan^{-1}\left(\frac{b}{a}\right)\\ \; & =\arctan^{-1}\left(\frac{r_{1}\sin\omega_{1}+r_{2}\sin\omega_{2}}{r_{1}\cos\omega_{1}+r_{2}\cos\omega_{2}}\right). \end{array}$$

According to the discussion above, Equation (12) can be transformed into

(A4) $$\begin{array}{cc} r_{c}\left(t\right) & =\left(\frac{\lambda_{c}}{4\pi d_{0}}\right)e^{j(-2\pi f_{d}t\cos\theta -\phi_{d})}\\ \; & +\left(\frac{\lambda_{c}\sigma }{4\pi (d_{1}+d_{2})}\right)e^{j(-2\pi f_{r}t\cos\varphi -\phi_{r})}. \end{array}$$

So, the amplitude value (complex envelope) of $r_{c}\left(t\right)$ is

(A5) $$\begin{array}{cc} \; & |r_{c}\left(t\right)|\\ \; & =({\left(\frac{\lambda_{c}}{4\pi d_{0}}\right)}^{2}+{\left(\frac{\lambda_{c}\sigma }{4\pi (d_{1}+d_{2})}\right)}^{2}+2\frac{\lambda_{c}}{4\pi d_{0}}\cdot \frac{\lambda_{c}\sigma }{4\pi (d_{1}+d_{2})}\\ \; & \cdot \cos\left(2\pi \left(f_{r}\cos\varphi -f_{d}\cos\theta \right)t+\left(\phi_{r}-\phi_{d}\right)\right){)}^{\frac{1}{2}}\\ \; & =\frac{\lambda_{c}}{4\pi }(\frac{1}{d_{0}^{2}}+{\left(\frac{\sigma }{d_{1}+d_{2}}\right)}^{2}+\frac{2\sigma }{d_{0}\left(d_{1}+d_{2}\right)}\\ \; & \cdot \cos\left(2\pi \left(f_{r}\cos\varphi -f_{d}\cos\theta \right)t+\left(\phi_{r}-\phi_{d}\right)\right){)}^{\frac{1}{2}}, \end{array}$$

and then the phase of $r_{c}\left(t\right)$ is given by

(A6) $$\begin{array}{cc} \; & ∠r_{c}\left(t\right)\\ \; & =\arctan^{-1}\left(-\frac{\frac{\lambda_{c}}{4\pi d_{0}}\sin\left(\Lambda +\phi_{d}\right)+\frac{\lambda_{c}\sigma }{4\pi (d_{1}+d_{2})}\sin\left(\Gamma +\phi_{r}\right)}{\frac{\lambda_{c}\sigma }{4\pi d_{0}}\cos\left(\Lambda +\phi_{d}\right)+\frac{\lambda_{c}}{4\pi (d_{1}+d_{2})}\cos\left(\Gamma +\phi_{r}\right)}\right), \end{array}$$

where $\Lambda =2\pi f_{d}t\cos\theta$ and $\Gamma =2\pi f_{r}t\cos\varphi$.

When the constant phase term, ϕ, is an integer multiple of 2π, Equations (A5) and (A6) simplify, respectively, to

(A7) $$\begin{array}{cc} \; & |r_{c}\left(t\right)|\\ \; & =({\left(\frac{\lambda_{c}}{4\pi d_{0}}\right)}^{2}+{\left(\frac{\lambda_{c}\sigma }{4\pi (d_{1}+d_{2})}\right)}^{2}+2\frac{\lambda_{c}}{4\pi d_{0}}\cdot \frac{\lambda_{c}\sigma }{4\pi (d_{1}+d_{2})}\\ \; & \cdot \cos\left(2\pi \left(f_{r}\cos\varphi -f_{d}\cos\theta \right)t+\left(\phi_{r}-\phi_{d}\right)\right){)}^{\frac{1}{2}}\\ \; & =\frac{\lambda_{c}}{4\pi }(\frac{1}{d_{0}^{2}}+{\left(\frac{\sigma }{d_{1}+d_{2}}\right)}^{2}+\frac{2\sigma }{d_{0}\left(d_{1}+d_{2}\right)}\\ \; & \cdot \cos\left(2\pi \left(f_{r}\cos\varphi -f_{d}\cos\theta \right)t\right){)}^{\frac{1}{2}}, \end{array}$$

and

(A8) $$\begin{array}{c} ∠r_{c}\left(t\right)=-\arctan^{-1}\tan\left(\frac{\left(d_{1}+d_{2}\right)\sin\Lambda +\sigma d_{0}\sin\Gamma }{\left(d_{1}+d_{2}\right)\cos\Lambda +\sigma d_{0}\cos\Gamma }\right). \end{array}$$
